# Comparison of single- and multi-trait approaches to identify best wild candidates for aquaculture shows that the simple way fails

**DOI:** 10.1038/s41598-020-68315-5

**Published:** 2020-07-14

**Authors:** Lola Toomey, Thomas Lecocq, Zoltán Bokor, Laurent Espinat, Árpád Ferincz, Chloé Goulon, Sami Vesala, Margot Baratçabal, Mamadou-Diouhe Barry, Mélanie Gouret, Camille Gouron, Ádám Staszny, Emilie Mauduit, Vicheka Mean, Iris Muller, Nicolas Schlick, Kévin Speder, Romain Thumerel, Clémentine Piatti, Alain Pasquet, Pascal Fontaine

**Affiliations:** 1 University of Lorraine, INRAE, URAFPA, 54000 Nancy, France; 2 Department of Aquaculture, Szent István University, Gödöllő, Hungary; 3 Université Savoie Mont Blanc, INRAE, UMR CARRTEL, 75 bis Avenue de Corzent, CS 50511, 74200 Thonon-les-Bains cedex, France; 4 Natural Resources Institute Finland, Helsinki, Finland; 5 CNRS (Centre National de la Recherche Scientifique), Paris, France

**Keywords:** Zoology, Animal behaviour, Ichthyology, Biodiversity, Ecosystem services, Sustainability

## Abstract

In agriculture, diversifying production implies picking up, in the wild biodiversity, species or populations that can be domesticated and fruitfully produced. Two alternative approaches are available to highlight wild candidate(s) with high suitability for aquaculture: the single-trait (i.e. considering a single phenotypic trait and, thus, a single biological function) and multi-trait (i.e. considering multiple phenotypic traits involved in several biological functions) approaches. Although the former is the traditional and the simplest method, the latter could be theoretically more efficient. However, an explicit comparison of advantages and pitfalls between these approaches is lacking to date in aquaculture. Here, we compared the two approaches to identify best candidate(s) between four wild allopatric populations of *Perca fluviatilis* in standardised aquaculture conditions. Our results showed that the single-trait approach can (1) miss key divergences between populations and (2) highlight different best candidate(s) depending on the trait considered. In contrast, the multi-trait approach allowed identifying the population with the highest domestication potential thanks to several congruent lines of evidence. Nevertheless, such an integrative assessment is achieved with a far more time-consuming and expensive study. Therefore, improvements and rationalisations will be needed to make the multi-trait approach a promising way in the aquaculture development.

## Introduction

The emergence of agriculture is one of the most important evolutions in human history. It was enabled by wild species domestication^1^. Domestication is the process in which groups of individuals are bred in a human-controlled environment and modified across succeeding generations from their wild ancestors, in ways these become more useful to humans who increasingly control their food supply and reproduction^2^. This process ranges from the first trials of acclimatisation to the setting up of selective breeding programmes^3^. The main wave of domestication for fishes only started at the beginning of the twentieth century to develop aquaculture (i.e. the farming of aquatic organisms), notably to mitigate provisioning service disruptions due to fishery collapse^3^. Aquaculture is the fastest-growing food production sector in the world and now provides about 50% of the world's aquatic food consumption^3^. However, the aquaculture development has been criticised, notably because of its negative consequences on environments and its potential unsustainable development^3,4,6^. Despite the numerous attempts to domesticate new fish species, one of the main weaknesses of today’s aquaculture is its low species diversity (i.e. 85% of the world fish production relies on about fifteen species^5^). Indeed, this latter threatens (1) wild native fauna (e.g. biological invasions, pathogen spill over^7–9^), (2) food security (e.g. epizooty hazard^10^), and (3) economic prospect (e.g. low adaptive potential to face environment/market fluctuations)^4^. Therefore, one of the ways promoted to ensure the sustainability of the aquaculture sector relies on the production diversification^3,4^. Although diversification effort in the last ten years has already made aquaculture production far more diverse than the terrestrial animal agriculture, this trends is expected to continue in the next decades to further increase aquaculture sustainability, as well as to answer to the decline of wild fish stocks.

Promoting species production diversification is not an easy task since it implies domesticating and commercially producing new species. This often fails due to either technical limitations, economic constraints, or intrinsic species features^3,11,12^ (see also for non-fish species^1,13,14^). These latter features are, for instance, low growth rate, high aggressiveness level, or low tolerance to high rearing densities, which are seen as impeding traits to start fruitful species production^11,12,15^. On the opposite, the ability to grow, feed, reproduce, and tolerate conspecifics and aquaculture-induced stress facilitates domestication^11,13^. This means that the predisposition to be successfully domesticated and produced is species-dependant^11^. Furthermore, these features could also vary at the intraspecific level, especially due to geographic differentiation (e.g.^16,17^). Indeed, this differentiation between geographically distant conspecific populations^18^, triggered by distinct demographic histories and local adaptations, can shape population specificities in key features for domesticating and commercially producing fishes (e.g. growth and development^19,20^). Therefore, taking into account geographic differentiation could facilitate new species domestication^16,19^.

Evaluating the ability of a species/population to be successfully domesticated and commercially produced requires an initial assessment of its expression of key trait(s). Looking back at past domestication programmes, two alternative paradigms can be used to identify suitable species or population(s) for further production development: the (1) single-trait and (2) multi-trait approaches.

The single-trait assessment is the traditional approach which consists of studying a single phenotypic trait, and consequently a single biological function, to identify population(s) with desirable expression of the trait for further production development. This approach has been widely applied at the beginning or at advanced stages of the domestication process in agriculture of animals (e.g.^21–26^; see also for plants^27^). Single-trait assessments often focus on an easily measurable trait which expression is involved in the domestication process and/or production profitability. Most of the time, growth rate is considered as a premium criterion^22,28^. This was for instance the case with Nile tilapia (*Oreochromis niloticus*), which domestication programmes initially focused on the growth rate^25^ (see for other species, e.g. ^28–29^). However, other traits are sometimes considered. For example, the beginning of the silver fox (*Vulpes fulvus*) domestication programmes focused on tameness^23,30^ (which facilitates the domestication process^1,11^). In the same way, selective breeding programmes in land agriculture have often focused on fertility^21^. Therefore, there is no absolute consensus about which trait should be chosen to perform the single-trait approach since various traits were previously used.

The multi-trait approach has recently been raised as an alternative method to the single-trait assessment. It has been suggested as a promising way to overcome domestication bottlenecks (e.g.^22,24,31,32^). Such approaches have been used in aquaculture for species at the beginning of domestication process but often only to a certain extend. For instance, multi-trait approaches were developed but the set of features considered reflected only one biological function (e.g. growth and production quality in^33^). Yet, a successful domestication process requires the favourable expression of several traits involved in various biological functions. Indeed, it is possible to successfully domesticate a population if it can reproduce, feed, grow, and overcome stresses in a human-controlled environment^22,31^. First applied in land agriculture (e.g.^31^), this approach is now promoted in fish aquaculture, especially at advanced stages of domestication, for selective breeding programmes^22,24,28^. At these advanced stages, growth performance, morphology, disease resistance, flesh quality traits, age at sexual maturation, and feed efficiency have been considered^22,24^. However, it is still poorly considered at the beginning of the domestication processes (i.e. first trials of acclimatisation of new candidates) even though a few examples exist (population^34^ or species selections^35^).

Although the multi-trait approach is the focus of an increasing research in aquaculture, an explicit assessment of its advantages and its pitfalls has not been performed to date. Indeed, no comparison between single-trait and multi-trait approaches to highlight best wild populations for fish aquaculture is currently available. Here, we aim at providing the first assessment of consequences of using single-trait or multi-trait approaches in aquaculture development. We compare the potential suitability for aquaculture of four wild allopatric populations of a test-case species, the European perch (*Perca fluviatilis*), during the larval period using the two different paradigms. We hypothesise that these two approaches lead to divergent conclusions.

## Results

**Figure 1:**
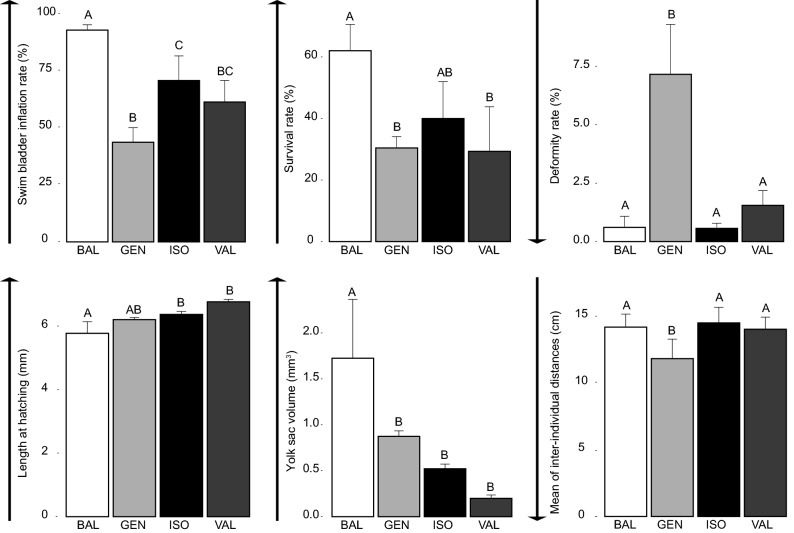
Barplots representing results obtained for traits studied in phase I for which a statistically significant difference was found between populations (n = 3 per population except for activity and inter-individual distances for which n = 9). Different letters indicate significant differences between populations (p value < 0.05) using post-hoc tests. The arrow represents how the expression of each trait should vary to meet stakeholder demands.

**Figure 2:**
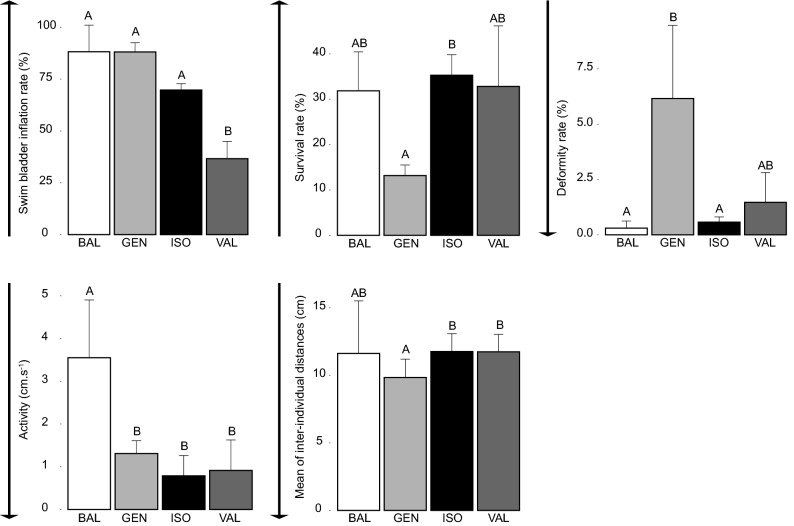
Barplots representing results obtained for traits studied in phase II for which a statistically significant difference was found between populations (n = 3 per population except for activity and inter-individual distances for which n = 9). Different letters indicate significant differences between populations (p value < 0.05) using post hoc tests. The arrow represents how the expression of each trait should vary to meet stakeholder demands.

We studied four wild lakes (Appendix S1): Valkea-Müstajärvi (VAL), Iso-Valkjärvi (ISO), Geneva (GEN), and Balaton (BAL). We compared populations at the larval stage based on 12 traits involved in growth, development, nutrition, and behaviour (Figs. 1, 2). These traits can impact the ability to be domesticated and fruitfully produced in aquaculture. For the single-trait approach, we considered all possibilities of initial trait choice. This means that we performed independent assessments of aquaculture suitability based on each trait independently. For the multi-trait approach, we achieved an integrative assessment by using a domestication potential index (Table 1; Appendix S2) to reach a consensus between results observed for all traits considered. The calculation of the domestication potential score was possible thanks to a survey addressed to perch farmers, which allowed to assign to each trait an average weighting coefficient (i.e. level of importance according to perch farmers). Correlations between traits were evaluated in order to determine if there were redundant traits in the multi-trait assessment. All details are in the “Material and methods” section.

**Table 1:** Domestication potential score obtained for the four *Perca fluviatilis* wild populations in the multi-trait analysis.

	ISO	VAL	GEN	BAL
Domestication potential score	37.898	35.089	34.667	57.166
Domestication potential rank	2	3	4	1

*ISO* Lake Iso-Valkjärvi, *VAL* Lake Valkea-Müstajärvi, *GEN* Lake Geneva, *BAL* Lake Balaton.

### Inter-populational differences in phase I

No statistical difference between populations was found for final length heterogeneity (F_(3,8; where 3 corresponds to the first degree of liberty [number of populations−1] and 8 to the second degree of liberty [total number of replicates−number of populations])_ = 1.27, p value = 0.35) and activity (F_(3,32)_ = 2.79, p value = 0.06).

A significant statistical difference between populations was found for the swim bladder inflation rate (F_(3,8)_ = 19.79, p value = 4.65 × 10^–4^), survival rate (F_(3,8)_ = 7.29, p value = 0.019), deformity rate (F_(3,8)_ = 22.88, p value = 2.80 × 10^–4^), length at hatching (F_(3,8)_ = 12.45, p value = 2.87 × 10^–3^), yolk sac volume (F_(3,8)_ = 10.85, p value = 2.21 × 10^–3^), and for the mean of inter-individual distances (F_(3,32)_ = 10.22, p value = 7.12 × 10^–5^) (Fig. 1).

### Inter-populational differences in phase II

No statistical difference between populations was found for specific growth rate in length (F_(3,8)_ = 2.20, p value = 0.17) and weight (F_(3,8)_ = 3.06, p value = 0.09), final growth heterogeneity for length (F_(3,8)_ = 1.26, p value = 0.35) and weight (F_(3,8)_ = 0.49, p value = 0.70), and aggressiveness (F_(3,8)_ = 2.58, p value = 0.15).

A statistically significant difference was found between populations (Fig. 2) for swim bladder inflation rate (F_(3,8)_ = 22.97, p value = 1.36 × 10^–4^), survival rate (F_(3,8)_ = 4.60, p value = 0.040), deformity rate (F_(3,8)_ = 6.95, p value = 0.013), activity (F_(3,32)_ = 15.77, p value = 1.79 × 10^–6^), and the mean of inter-individual distances (K = 8.25, df = 3, p value = 0.041) (Fig. 2).

### Evaluation of the population suitability for domestication

Regarding the single-trait approach, populations’ suitability for aquaculture differed depending on the trait initially chosen and were not consistent across experimental phases (Figs. 1, 2). For instance, when considering only growth rate, it did not allow identifying a differentiation in the suitability for domestication between populations. On the contrary, when considering deformity rate, GEN appeared as the least suitable population in both phases. In the same way, when considering swim bladder inflation rate, BAL presented the highest suitability for domestication in phase I but that would hide the fact that this population presented in phase II the highest activity rate (Figs. 1, 2). Regarding the multi-trait approach, it highlighted that the population with the highest domestication potential score is BAL, followed by ISO, VAL, and GEN (Table 1; Appendix S2).

### Phenotypic correlations

Very few correlations were detected: 14 pairs of traits were positively correlated (p value < 0.05), 13 pairs were negatively correlated (p value < 0.05), and 144 pairs were not significantly correlated (Appendix S3).

## Discussion

### Limitations of the population performance evaluation

Our results were based on a common garden experiment which allows investigating the genetic basis of phenotypic differences (i.e. heritable divergences) between populations^36^. Therefore, it is an efficient way to compare larviculture performances between several wild populations. However, some limitations must be underlined. Indeed, evaluations were based on individuals originating from eggs collected from the wild. Therefore, we cannot completely rule out that some of the observed trait expressions had been shaped by phenotypic plasticity (i.e. the ability of a genotype to display several phenotypes when exposed to different environments^37^) or transgenerational effects^38^. Therefore, aquaculture performances of the four studied European perch populations should be considered with caution by fish farmers. Nevertheless, we argue that we minimised these potential biases thanks to our common-garden environment. Moreover, if such limitations ever impact our results, they would affect both the single-trait and the multi-trait approaches. Finally, this study aimed at comparing the two approaches rather than highlighting the best biological unit for larviculture within *P. fluviatilis.* Further comparisons between populations over the entire lifecycle using the multi-trait approach would allow identifying within species the best unit to further develop *P. fluviatilis* production.

### When the simple way fails

Our study shows that the alternative single-trait and multi-trait approaches can lead to divergent results when identifying the best population(s) for aquaculture purpose.

Overall, we detect three main limitations of the single-trait approach. First, considering a single trait can lead to neglect the potential available in the wild biodiversity if the targeted trait does not vary between studied populations. This is particularly noticeable for the growth rate (no significant differentiation between populations despite a differential length at hatching; Fig. 1), which has traditionally been the most considered trait when assessing performances in aquaculture^22,25^. In the worst-case scenario, this would lead to start a production with a suboptimal population that will have to be improved through costly and difficult selective breeding programmes while better candidates were available in the wild. Second, the identification of populations with the highest suitability for aquaculture can vary depending on the trait picked for evaluation. For instance, the observed differentiations for survival and deformity rates allow identifying best population(s) for aquaculture but do not converge to highlight the same best population(s) (Fig. 2). Furthermore, some of the conclusions obtained by the alternative tested single-trait approaches are completely contradictory (e.g. deformity rate would identify Lake Balaton as the one of the best populations while if activity is considered, it would qualify this population as the least suitable; Figs. 1, 2). This places a premium on the definition of the most important trait for fish production although this is still unachievable information due to divergent opinions between fish farmers and the numerous traits evaluated as very important (e.g. Appendix S2). Third, the choice of a population based on a single trait, and consequently a single biological function, can lead to start fish domestication/production with a population displaying deleterious expression of other key traits. Indeed, since traits are linked in complex ways, selection of only one trait will inevitably be associated with the indirect selection of other traits (i.e. through linkage and/or pleiotropy^39^), including undesirable characteristics^40,41^. Eventually, this leads to deleterious impacts of the species production^29^. The risk of co-selecting a deleterious trait which is not considered in the single-trait approach is a well-known fact in land species domestication history (but see for aquaculture negative genetic correlations, e.g.^42^). For instance, continuous selection towards milk yield and growth led respectively to a decrease of (1) fertility in dairy cow^21^ and (2) reproductive and immune performances in broilers^26^. In the same way, artificial selection in domestic turkeys for large breast size has led to the artificial insemination of females due to the inability of males to copulate naturally^43^. Similarly, the focus on taming at the beginning of the domestication process of the silver fox (*Vulpes fulvus*) led to morphological changes disadvantageous for the pelt market^23^.

### More is more but it is more complicated too

Multi-trait approach allows overcoming the single-trait method limitations. Indeed, investigating several traits minimises the risk of missing valuable populational specificities or choosing a population with hidden undesirable characteristics. Therefore, the nascent promotion of multi-trait approach in land and aquatic agriculture development^22,32,39^ is a timely and welcomed trend. Moreover, it provides a more efficient and realistic assessment to start new domestication processes and to increase the sector sustainability since they both involve several biological functions that could be studied only through the consideration of many traits. Indeed, as mentioned before, the domestication itself involves several biological functions such as growth, nutrition, behaviour, and reproduction. Furthermore, the willingness to develop a more sustainable aquaculture production implies for instance to study traits involved in robustness (e.g. resistance to thermal changes, vulnerability to new pathogens/parasites) and nutrition (e.g. ability to grow with a plant-based diet)^4^. However, beyond the bright sides of multi-trait approach, three main limitations could make its implementation difficult.

First, the most obvious pragmatic issue is that workload and cost associated with this approach are much more substantial than with the single-trait approach. One potential solution to facilitate the multi-trait assessment consists of restricting the assessment framework to a limited set of traits. This could be achieved through trait correlation evaluation. Some traits could be removed from the assessment framework by considering phenotypic correlations between traits (assuming phenotypic correlations are a good proxy for genetic correlations^44,45^). Indeed, when such correlations are observed (e.g. feed efficiency improvement when growth rate considered^24,46^, aggressiveness decrease when growth rate considered^47^), one could expect that by focusing on a few traits, it is possible to take into account expressions of other correlated traits. However, in our study, we detected only few correlations including biological non-relevant relationships (e.g. positive correlation between length at hatching and activity in phase II). In this work, trait correlations are not helpful to restrict the evaluation to a handful of traits. Nevertheless, the complexity of multi-trait assessment should be seen as balanced by choosing the best part of the wild biodiversity before starting large-scale domestication programmes which are far more expensive and time-consuming.

Second, methodological limitations could impede the relevance of the multi-trait and multi-function approach. Indeed, the lack of efficient methods to measure some traits (e.g. assessment of traits involved in the nutrition function such as food conversion ratio requiring complex protocol^48^) and the increased risk of errors when working on multiple traits could lead to false decisions when choosing candidates for domestication/production. However, research advances are making (1) fish trait information available or (2) trait analysis methods easier and cheaper.

Finally, working with multiple traits implies using a method to make a consensus between results obtained for individual traits. Here, we proposed a method, which allows attributing a score to each population in order to identify the best candidate(s) for aquaculture within species. It is worth nothing that this scoring method does not reflect the differentiation degree between populations. Indeed, ranks are used to classify populations, but these ranks do not show how much differentiated populations are (see “Material and methods” section for calculation details; Appendix S2). However, it allows putting all traits at the same level and then uses weighting coefficient to take into account trait importance divergences according to fish farmers. Other alternative methods could have been used but, nowadays, there is no consensus about which method should be favoured (e.g. using the majority rule: the best population is the one which is ranked first for a majority number of traits; it would also highlight Lake Balaton as the best population but does not take into account divergent levels of importance of the traits; Appendix S2).

## Conclusion

Our results showed that the single-trait approach can (1) miss key divergences between the populations and (2) highlight different best candidate(s). Conversely, the multi-trait approach, which includes several key traits for larviculture, allowed identifying the population with the highest domestication potential thanks to several congruent lines of evidence. Nevertheless, this approach is more complex and requires making consensus between trait results. However, despite these limitations, it appears as the most suitable approach to identify within species populations with higher domestication potential.

## Material and methods

### Test-case species

*Perca fluviatilis* (Actinopterygii, Percidae) is a common widely spread Eurasian species living in freshwater and brackish habitats^49^. Its economic (high market value) and recreational (i.e. fishing) interests have led to the development of its RAS (i.e. recirculating aquaculture systems) farming since the 1990’s^49^. *Perca fluviatilis* is among the most interesting species for the development of inland aquaculture in Europe^49^. However, despite its economic potential, the production is still limited. This is mainly due to major bottlenecks, especially in first-life stages, such as low growth rate, low survival rate, and high cannibalism rate^49^. This highlights the potential interest of re-starting new domestication programmes. An intraspecific differentiation was already showed for this species in standardised conditions (e.g. growth^19,50^, behaviour^16^, development^51^).

### Biological material collection and pre-experiment rearing conditions

Population sampling and rearing conditions were adapted from Toomey et al.^16^. Egg ribbons (one ribbon corresponding to one female) were obtained during the May 2018 and May 2019 spawning seasons from four lakes (Appendix S1): Valkea-Müstajärvi (VAL; 2018; Finland; 61° 13′ 08″ N, 25° 07′ 05″ E), Iso-Valkjärvi (ISO; 2018; Finland; 60° 57′ 21″ N, 26° 13′ 3″ E), Geneva (GEN; 2019; France; 46° 22′ 7.20″ N, 6° 27′ 14.73″ E), and Balaton (BAL ; 2019 ; Hungary ; 46° 54′ 23.375″ N, 18° 2′ 43.119″ E). We chose these populations since a phenotypic differentiation is known between the Finnish and Geneva populations^16^ while genetic specificities of central Europe populations have been already observed^52,53^. After transportation, 13–19 egg ribbons per lake were incubated at the experimental platform of aquaculture (Unit of Animal Research and Functionality of Animal Products, University of Lorraine, Vandœuvre-lès-Nancy, France) at 13 °C in incubators (110 × 64 × 186 cm; one population per incubator to avoid potential disease transmission between populations, one to two incubators per population) containing nine racks each (45 × 7 × 12 cm). Each incubator had its own temperature control and recirculated water system (flow rate of 4 m^3^ h^−1^). Water was UV-sterilized. Temperature (13.0 ± 0.4 °C) and oxygen rate (10.0 ± 0.5 mg L^−1^) were checked daily while pH (8.0 ± 0.2) was monitored three times a week (± standard error). Ammonium and nitrite concentrations (lower than 0.05 mg L^−1^) were measured three times a week until hatching. Light intensity was 400 lx at the water surface. Photoperiod was 12L:12D (12 h of light and 12 h of darkness).

### Experimental rearing protocol

Two independent experimental phases were performed: phase I from hatching until the end of weaning (i.e. transition from live feed to inert pellets; 26 days post-hatching, dph) and phase II from 27 dph until the end of nursery, at 60 dph. The larval period was split in two phases in order to ensure availability of larvae across the whole larval period since there is a very high mortality rate during first-life stages. Because wild egg ribbons are not available the same time for all populations (i.e. asynchronous spawning seasons) and in order to prevent potential pathogen transmission, all populations were reared in independent structures. Since there are increasing concerns about the epizootic disease Perhabdovirus in Europe^49^, all populations were tested for the occurrence of this virus (*Laboratoire Département d’Analyses du Jura*, Poligny, France). All results were negative to the presence of the Perhabdovirus.

Regarding phase I, larvae from the different egg ribbons of each population were mixed after hatching and transferred to three green (RGB: 137, 172, 118) internal-wall 71 L cylindro-conical tanks (three replicates per population; RAS) at a density of 50 larvae L^−1^. Photoperiod was 12L:12D (simulation of dawn and dusk for 30 min) and light intensity was 400 lx at the water surface. Temperature was gradually increased during the first 2 weeks to 20 °C (1 °C day^−1^). Larvae were hand-fed with newly hatched *Artemia* nauplii (Sep-Art, INVE; seven times a day, every 1 h 30 from 8.30 am to 5.30 pm) from 3 days post-hatching until at 16 dph, which corresponds to the beginning of the weaning (i.e. transition from live feed to inert dry artificial diet) period. At 16 dph, *Artemia* ration was decreased by 25% every 3 days and dry feed ration (BioMar, 300 µm until 21 dph, then 500 µm) was increased by the same ratio. After 25 dph, larvae were fed with dry feed ad libitum (BioMar 500 µm, then 700 µm at 44 dph until 60 dph). At 26 dph, the larvae left in the cylindro-conical tanks were removed in order to start phase II.

Regarding phase II, after hatching, larvae left (i.e. not sampled for phase I) were held in 2 m^3^ tanks (RAS). The same conditions as phase I were used (temperature, light intensity, feeding, and photoperiod regimes). At 27 dph, these larvae were transferred to the three cylindro-conical tanks in order to start phase II at a density of 19 larvae L^−1^. Light intensity was 80 lx at water surface, all else remaining the same as phase I (except for density).

During the two phases, oxygen concentration (8.5 ± 2.3 mg L^−1^) and temperature (20.0 ± 0.6 °C) were checked daily in all tanks (± standard error). Ammonium and nitrite concentrations (means inferior to 0.05 mg L^−1^) and pH (7.7 ± 0.6 mg L^−1^) were monitored three times a week (± standard error). Tanks were cleaned daily after first feeding and dead individuals were removed every morning.

### Larviculture performance assessment

A trait is considered in this study at the replicate level. Each trait value is obtained from the mean of individual values.

#### Survival and development traits

Survival rate is one of the key traits contributing to the success of larviculture production^49,54^. Because of fast decomposition of dead larvae, it was not possible to count dead larvae during the first 5 days post-hatching. Consequently, the daily count of dead larvae was only performed in phase II. Therefore, survival rate in phase I was calculated for each cylindro-conical tank thanks to the final count of larvae using the following formula: Nf × 100/(Ni − Ns), where Nf is the final number of fish counted at the end of phase I, Ni the initial number of fish, and Ns the number of fish sampled along the phase (i.e. for behaviour experiments, see below). For phase II, the Bergot survival rate^55^ was used since it takes into account the number of fish removed for sampling and the daily mortality. Two traits related to the development of individuals and essential for larviculture were considered^49^: swim bladder inflation rate and deformity rate. Swim bladder inflation rate was estimated at the end of each experimental phase (following the protocol used in Jacquemond^56^; 20 g L^−1^ of sea salt and 70 mg L^−1^ of MS-222): 100 × (SB + /Nf) with SB + the number of larvae with swim bladder and Nf the final number of larvae. Deformity rate was estimated in the final counting of each experimental phase using the following formula: 100 × (Nm/Nf) with Nm the number of deformed larvae (only visible column deformities) and Nf the final number of larvae counted. Finally, the volume of the yolk sac was also evaluated at 1 day post-hatching since it reflects the quantity of nutritional reserves available before exogenous feeding^49^. It is calculated using the following formula: π/6 × YSL×YSH^2^, where YSL is the length of the yolk sac and YSH the height of the yolk sac^57^.

#### Behavioural traits

The ability of a biological unit to be efficiently produced in intensive conditions (i.e. intensive farming is an increasing trend in the aquaculture development) also depends on (1) inter-individual relationships, (2) inter-individual distances, and (3) activity^16^. Indeed, tolerance to conspecifics in a restricted area is essential for production since it can impact individual welfare^58^. Highlighting populations which present a cohesive group structure would be advantageous. Nevertheless, living in group is not costless because it can trigger aggressive behaviours which can lead to uneven competition for food, mortalities, stress, or immune suppression^16^. Therefore, both inter-individual distances and inter-individual interactions need to be considered. Finally, activity is also important since it contributes to the total energetic budget^16^. Furthermore, less active individuals could contribute to decrease the occurrence of inter-individual contacts and subsequent potential aggressive interactions.

Regarding aggressive interaction quantification, aggressiveness was calculated for phase II using the formula : 100 × (Ni − (Nf + Nd + Ns) + Nc)/(Nf − Ns) with Ni the initial number of larvae, Nf the final number of larvae, Nd the sum of dead larvae counted daily, Ns the number of sampled larvae, and Nc the number of truncated or enucleated (enucleation being a specific indicator of aggressiveness in perch^16^) larvae among the dead ones (not possible in phase I since it was not possible to count dead larvae during the first 5 days post-hatching due to fast decomposition; in addition, no truncated or enucleated individuals were recorded for phase I).

Regarding the evaluation of inter-individual distances and activity, the detailed protocol is available in Toomey et al.^16^. Briefly, for each population, three replicates for each cylindro-conical tank were performed (nine replicates per population) over 2 days for phase I (25 and 26 dph) and phase II (44 and 45 dph). For each population, 90 individuals (n = 30 per cylindro-conical tank, 10 individuals per replicate) were sampled and transferred to three aquaria (58 L; light intensity of 80 lx light intensity, 20 °C). After one night of acclimatisation, populations were tested per groups of ten individuals in circular arenas (30 cm diameter, 1.5 cm of water depth, 10 lx) to study inter-individual distances and activity^16^. After 30 min acclimatisation, individuals were filmed for 30 min. After the experiment, individuals were euthanized (overdose of MS-222) and kept in formalin 4% for later length measurements. Larvae tested in the circular arenas from ISO, VAL, BAL, and GEN were respectively 14.05 ± 0.55 mm, 12.90 ± 0.62 mm, 10.62 ± 0.47 mm, and 11.81 ± 1.01 mm during phase I and 26.74 ± 1.67 mm, 26.28 ± 1.99 mm, 19.24 ± 1.22 mm, and 12.26 ± 0.45 mm during phase II (± standard error). Analyses were performed using the ImageJ software^59^. Images were extracted from videos at 5-min interval (six images per video). For each image, coordinates of individuals were noted using the middle point between the eyes. The mean of inter-individual distances was evaluated per replicate. It corresponds to the mean of distances between a focal fish and all the other fish of the group and it is an indicator of the group cohesion. Detailed calculation is available in Colchen et al*.*^60^. Activity was analysed in ImageJ. Every 5 min, one image per second was extracted for six consecutive seconds. For each image, coordinates of each individual were noted. This allowed calculating distance swam every second during the 5 s. The mean allowed obtaining for each individual the mean distance swam per second. From these values, we were able to calculate an average activity per replicate.

#### Growth traits

Growth traits are important in larviculture production, more particularly the length at hatching, specific growth rate, and growth heterogeneity^49^. To evaluate these traits, 30 larvae per population (i.e. ten larvae per cylindro-conical tank) were sampled the first and last days of each experimental phase, euthanized with an overdose of MS-222, and kept in formalin 4%. For phases I and II, larvae were measured using ImageJ (± 0.01 mm). For phase II, larvae were also individually weighted (± 0.1 mg; not possible in phase I due to the imprecision of measure at 1 day post-hatching). Since specific growth rate (SGR) is a trait of interest at the end of larviculture, it was calculated only in phase II using the following formula: SGR = 100 × (ln(Xf) − ln(Xi)) × ∆T^−1^ where Xi and Xf are respectively the average initial and final weight/length and ∆T the duration of phase II. Final growth heterogeneity was calculated for both phases in the following way: CV_Xf_/CV_Xi_ in which CV is the coefficient of variation (100 × standard deviation/mean) and Xi and Xf the initial and final weight/length, respectively.

### Statistical analyses

All statistical analyses were performed in R 3.0.3^61^ to assess if there were statistical differences (p value < 0.05) in traits between populations. To test the normality of distribution, a Shapiro–Wilk test was used. Homogeneity of variances was tested using the Levene test (R-package *lawstat*). When assumptions were not met, data was log-transformed. Then, in order to check if the cylindro-conical tank had no influence on our results, Corrected Akaike Information Criterion (AICc; R-package *qpcR*) were used to compare linear mixed models (biological traits as fixed factors and cylindro-conical tanks as random factor; R-package *lmer*) and linear model (biological traits as fixed factors, no random factor). For most factors, there was no influence of the cylindro-conical tank on the model. Therefore, one-way analyses of variance (ANOVA F test) followed by Tukey post hoc tests were used to evaluate differences between populations. When the effect of the cylindro-conical tank was significant, the ANOVA was performed on the linear mixed model and estimated marginal means were calculated (R-package *emmeans*). When assumptions were not respected despite log-transformation (only for inter-individual distances in phase II), Kruskal–Wallis H test was used followed by Dunn post-hoc test (R-package *PMCMR*). All post-hoc results were corrected relatively to the number of comparisons using Benjamini–Hochberg procedure. In order to improve the multi-trait approach, we assessed if there were redundant traits in the assessment. To do so, Pearson’s correlation coefficients between traits were calculated (except for correlations with inter-individual distances in phase II for which Spearman’s correlation coefficients were calculated).

### Evaluation of the population suitability for domestication with the two approaches

Regarding the single trait analysis, growth rate is the most often considered trait in domestication programmes. However, in this study, we considered all possibilities of initial trait choice and analysed results for all alternative cases. This means that we obtained independent assessments of aquaculture suitability based on each trait independently. Providing a statistical difference is observed, we considered as the best population(s), the one (those) which displayed the most desirable expression (from a fish farmer point of view^49^). The expression value considered is the mean obtained over the three replicates.

In the multi-trait approach, an integrative decision framework is necessary in order to make a consensus between the different traits. Indeed, it is unlikely that a population has the best performances for all criteria. It is more likely that a unit displays a good performance for a specific trait (e.g. high growth rate) but appears as less valuable when considering another trait (e.g. low larval survival rate). Therefore, an indicator is required in order to make a synthesis at the multi-function and multi-trait levels to identify units with high domestication potential. Some methods and associated scores were suggested at the interspecific level in order to identify good candidate species (see for instance method used in^15,35^). However, previous scoring methods integrate some traits for which intraspecific variability is unlikely (e.g. presence of bones in Quémener et al*.*^15^) or do not include all traits considered here in the multi-function and multi-trait approach. Therefore, we propose a domestication potential score that aims at making a synthesis at the multi-trait level. The first step of this score calculation consists in ranking populations according to their performance for each trait (average rank per trait obtained from the mean of all replicate ranks; Appendix S2) when a statistical difference between populations was highlighted. Then, since all traits do not present the same level of importance for production according to fish farmers (which was confirmed in a survey we led before this work; see Appendix S2), it is necessary to adjust the importance given to each trait through the use of weighting coefficients (between zero and 100; adapted from Quémener et al*.*^15^, similarly to breeding goals index but for which each trait is weighted according to its socio-economic value^24^). Thanks to the survey addressed to perch farmers, we were able to assign to each trait an average weighting coefficient (Appendix S2). This survey also confirmed that traits studied were regarded as relevant by most questioned fish farmers (Appendix S2). For each trait, we then divided the average weighting coefficient attributed by fish farmers by the rank of the population (Appendix S2). When traits were evaluated over two phases, they were considered as two separate traits in the calculation of the domestication potential score. Once this ratio is attributed to each trait, the sum of all ratios allows calculating the domestication potential score for each population (Appendix S2). Overall, the domestication potential score (ranging from 0 to ∞) is defined as:

$$ {\text{Domestication potential score}} = \sum\limits_{{i = 1}}^{n} {\left( {\frac{{{\text{Weighting coefficient}}\left( {\text{i}} \right)}}{{{\text{Rank}}\left( {\text{i}} \right)}}} \right)}  $$

where n corresponds to the number of traits, *i* = 1 the first trait evaluated, *i* the trait considered, *n* = the last trait considered, and the weighting coefficient corresponds to the weight attributed to each trait by perch farmers and the rank corresponds to the rank attributed to the population for each trait.

The population with the highest score is the population with the highest potential for domestication.

### Ethical standards

All along this experiment, individuals were handled as little as possible. All procedures used in the experiment were in accordance with national and international guidelines for protection of animal welfare (Directive 2010/63/EU). This study was conducted with the approval Animal Care Committee of Lorraine (CELMA no. 66) and the French Ministry of Higher Education, Research, and Innovation (APAFIS13368-2018020511226118, APAFIS17164-2018101812118180).

## Supplementary information

Supplementary information

## Data Availability

All data generated or analysed during this study are included in this article (and its Supplementary Information files).
