# Supplementary material for: Comparison of single- and multi-trait approaches to identify best wild candidates for aquaculture shows that the simple way fails
Source: Sci Rep. 2020 Jul 14;10:11564. doi: 10.1038/s41598-020-68315-5 (PMC7360571; doi:10.1038/s41598-020-68315-5)
Supplement: Supplementary file 1 — Supplementary information [file 41598_2020_68315_MOESM1_ESM.pdf]

Comparison of single- and multi-trait approaches to identify best wild candidates for aquaculture shows that the simple way fails

Toomey Lola, Lecocq Thomas, Bokor Zoltán, Espinat Laurent, Ferincz Árpád, Goulon Chloé, Vesala Sami, Baratçabal Margot, Barry Mamadou-Diouhe, Gouret Mélanie, Gouron Camille, Staszny Ádám, Mauduit Emilie, Mean Vicheka, Muller Iris, Schlick Nicolas, Speder Kevin, Thumerel Romain, Piatti Clémentine, Pasquet Alain, Fontaine Pascal

Appendix S1: Map representing the four wild *Perca fluviatilis* populations sampled. VAL: Valkea-Müstajärvi, ISO : Iso-Valkjärvi, GEN : Geneva, and BAL: Balaton.

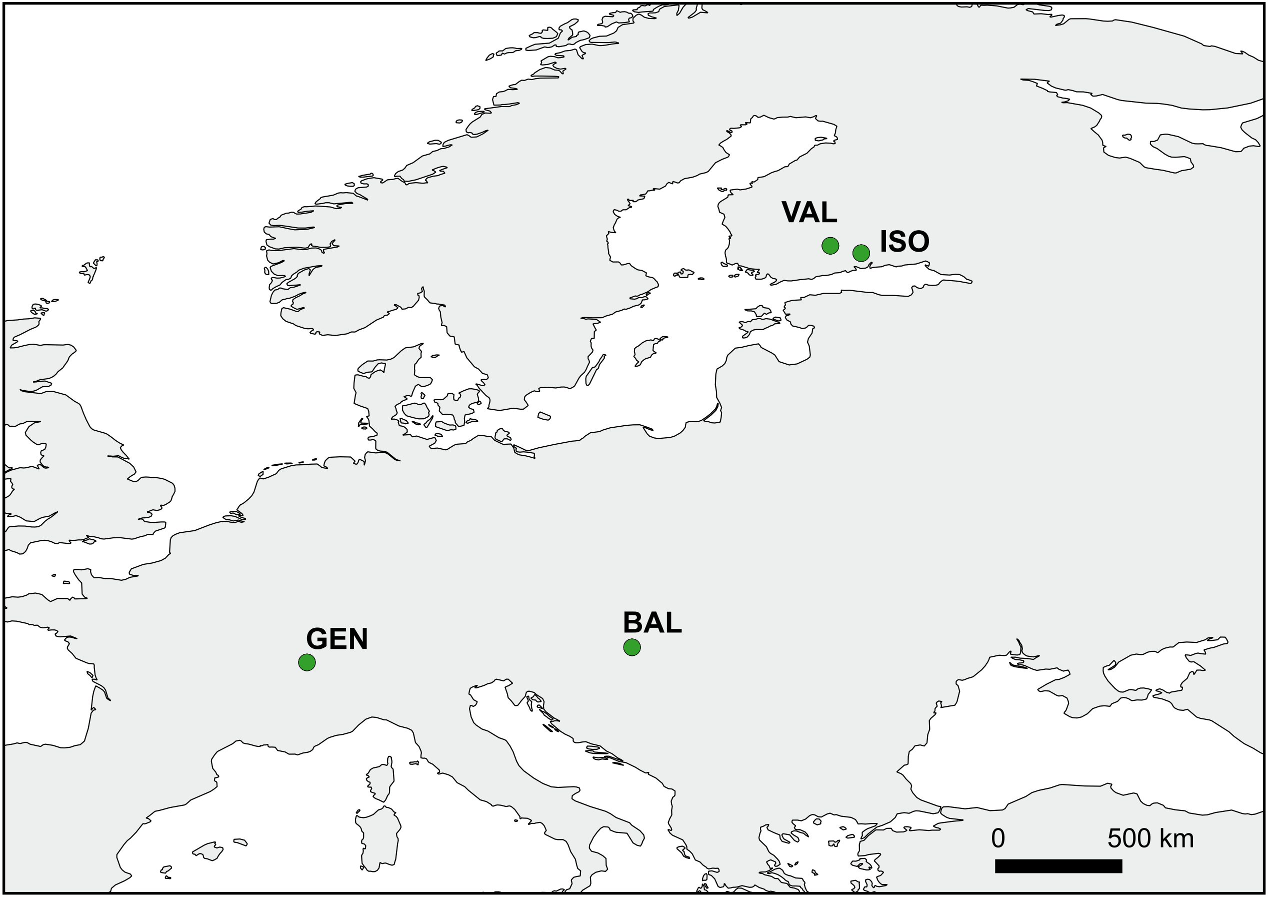

Appendix S2: A survey was performed between February and March 2019. Nine Percid farmers working with perch larviculture were questioned. We asked them to give their opinion on the importance of different traits involved in larval stages (from "not important" to "very important"). A weight coefficient was attributed to each opinion category: 0 for "not important", 25 for "slightly important", 50 for "Important", 75 for "Fairly important", and 100 for "very important". The first step consists in calculating an average weight coefficient for each trait taking into account farmers' opinions (step A). Then, for each replicate, populations are ranked per trait when a significant statistical differentiation was highlighted (p-value<0.05; Step B). This allows calculating an average rank per population and per trait (Step B). Finally, a score per trait and per population is calculated by dividing the average weighting coefficient by the average rank (Step C). The sum of all these scores allows calculating for each population a domestication potential score.

Step A

Trait weight	Not important		Slightly Important		Important		Fairly important		Very important		Weight coefficient sum	Total number of answers	Average weight coefficient
	Number of answers	Weight coefficient	Number of answers	Weight coefficient	Number of answers	Weight coefficient	Number of answers	Weight coefficient	Number of answers	Weight coefficient			
Trait / value attributed		0		25		50		75		100			
Larval growth rate	0	0	1	25	1	50	3	225	4	400	700	9	77,78
Larval growth heterogeneity	0	0	0	0	0	0	0	0	8	800	800	8	100,00
Volume of the yolk sac	0	0	2	50	0	0	1	75	5	500	625	8	78,13
Larval size at hatching	1	0	1	25	1	50	1	75	4	400	550	8	68,75
Swim bladder inflation rate	1	0	0	0	0	0	2	150	5	500	650	8	81,25
Larval deformity rate	0	0	0	0	0	0	1	75	8	800	875	9	97,22
Larval survival rate	0	0	0	0	1	50	2	150	6	600	800	9	88,89
Aggressiveness	0	0	0	0	1	50	2	150	6	600	800	9	88,89
Group structure (ability for shoaling, gregariousness)	2	0	2	50	1	50	3	225	1	100	425	9	47,22
Activity (i.e., fish more or less active)	0	0	3	75	1	50	2	150	1	100	375	7	53,57

Step B

		Replicate 1				Replicate 2				Replicate 3				Average rank			
Phase	Rank	ISO	VAL	GEN	BAL	ISO	VAL	GEN	BAL	ISO	VAL	GEN	BAL	ISO	VAL	GEN	BAL
II	Larval growth rate - Weight	No significant differentiation															
II	Larval growth rate - Length	No significant differentiation															
I	Larval growth heterogeneity - length	No significant differentiation															
II	Larval growth heterogeneity - length & weight	No significant differentiation															
I	Volume of the yolk sac	3	4	2	1	3	4	2	1	3	4	2	1	3,00	4,00	2,00	1,00
I	Larval size at hatching	3	1	2	4	2	1	3	4	2	1	3	4	2,33	1,00	2,67	4,00
I	Swim bladder inflation rate	3	2	4	1	2	3	4	1	3	2	4	1	2,67	2,33	4,00	1,00
II	Swim bladder inflation rate	3	4	2	1	3	4	1	2	3	4	2	1	3,00	4,00	1,67	1,33
I	Larval deformity rate	1	3	4	2	1	3	4	2	2	3	4	1	1,33	3,00	4,00	1,67
II	Larval deformity rate	2	3	4	1	3	2	4	1	2	3	4	1	2,33	2,67	4,00	1,00
I	Larval survival rate	3	4	2	1	2	3	4	1	2	4	3	1	2,33	3,67	3,00	1,00
II	Bergot Larval survival rate	1	3	4	2	2	1	4	3	3	2	4	1	2,00	2,00	4,00	2,00
II	Aggressiveness	No significant differentiation															
I	Inter-individual distances	4	2	1	3	3	2	1	4	4	2	1	3	3,67	2,00	1,00	3,33
II	Inter-individual distances	2	3	1	4	3	4	1	2	4	3	1	2	3,00	3,33	1,00	2,67
I	Activity (i.e., fish more or less active)	No significant differentiation															
II	Activity (i.e., fish more or less active)	2	1	3	4	1	2	3	4	1	2	3	4	1,33	1,67	3,00	4,00

Step C

Phase	Trait	Trait weight	ISO rank	VAL rank	GEN rank	BAL rank	Score per trait - ISO	Score per trait - VAL	Score per trait - GEN	Score per trait - BAL
II	Larval growth rate - Weight	77,78								
II	Larval growth rate - Length	77,78								
I	Larval growth heterogeneity - length	100,00								
II	Larval growth heterogeneity - length & weight	100,00								
I	Volume of the yolk sac	78,13	3,00	4,00	2,00	1,00	26,04	19,53	39,06	78,13
I	Larval size at hatching	68,75	2,33	1,00	2,67	4,00	29,46	68,75	25,78	17,19
I	Swim bladder inflation rate	81,25	2,67	2,33	4,00	1,00	30,47	34,82	20,31	81,25
II	Swim bladder inflation rate	81,25	3,00	4,00	1,67	1,33	27,08	20,31	48,75	60,94
I	Larval deformity rate	97,22	1,33	3,00	4,00	1,67	72,92	32,41	24,31	58,33
II	Larval deformity rate	97,22	2,33	2,67	4,00	1,00	41,67	36,46	24,31	97,22
I	Larval survival rate	88,89	2,33	3,67	3,00	1,00	38,10	24,24	29,63	88,89
II	Bergot Larval survival rate	88,89	2,00	2,00	4,00	2,00	44,44	44,44	22,22	44,44
II	Aggressiveness	88,89								
I	Inter-individual distances	47,22	3,67	2,00	1,00	3,33	12,88	23,61	47,22	14,17
II	Inter-individual distances	47,22	3,00	3,33	1,00	2,67	15,74	14,17	47,22	17,71
I	Activity (i.e., fish more or less active)	53,57								
II	Activity (i.e., fish more or less active)	53,57	1,33	1,67	3,00	4,00	40,18	32,14	17,86	13,39

DOMESTICATION POTENTIAL SCORE

	ISO	VAL	GEN	BAL
FINAL SCORE	378,98	350,89	346,67	571,66
FINAL RANK	2	3	4	1

Appendix S3: Correlation matrix between all traits (I relative to phase I and II referring to phase II) based on Pearson's correlation coefficients (between -1 and 1; except for comparisons with an asterisk (*) for which Spearman's correlation coefficients were used). Correlation values are indicated for each test and statistically significant correlations (p-value<0.05) are highlighted in bold. The colour corresponds to the value of the correlation coefficient (red for positive coefficients, blue for negative coefficients).

	Survival rate II	Inflation rate I	Inflation rate II	Deformity rate I	Deformity rate II	SGR length I	SGR length II	SGR Weight II	Aggressiveness	Length heterogeneity I	Weight heterogeneity II	Length heterogeneity II	Length at hatching	Yolk sac volume	Activity I	Inter-individual distances I	Activity II	Inter-individual distances II
Survival rate I	0.39	**0.71**	0.41	−0.46	−0.47	−0.03	0.52	**0.65**	−0.55	−0.12	−0.06	0.3	−0.54	0.57	0.37	0.32	**0.71**	0.15*
Survival rate II		0.45	−0.42	**−0.8**	**−0.71**	**−0.59**	0.4	0.11	−0.17	0.1	0.48	0.31	0.17	−0.17	−0.21	**0.7**	0.06	0.33*
Inflation rate I			0.11	**−0.76**	**−0.61**	−0.15	**0.71**	**0.64**	−0.3	0.05	−0.05	0.3	−0.45	0.51	0.01	**0.64**	**0.61**	0.33*
Inflation rate II				0.35	0.25	**0.85**	0	0.47	−0.44	−0.26	−0.07	0.38	**−0.79**	**0.74**	0.53	−0.4	0.45	−0.34*
Deformity rate I					**0.78**	0.5	−0.54	−0.26	0.09	−0.21	−0.04	−0.24	−0.03	−0.01	0.41	**−0.84**	−0.18	−0.26*
Deformity rate II						0.4	−0.31	−0.21	−0.04	−0.28	−0.17	0.12	0.01	−0.07	0.33	**−0.89**	−0.27	−0.42*
SGR length I							−0.14	0.3	−0.04	−0.06	−0.02	0.29	−0.56	0.44	0.4	−0.44	0.08	−0.31*
SGR length II								**0.79**	0.02	0.37	0.18	0.5	−0.11	0.1	0.35	0.52	0.36	−0.11*
SGR weight II									−0.05	0.36	0.23	0.44	−0.37	0.35	0.43	0.29	0.36	−0.13*
Aggressiveness										0.56	0.06	−0.39	**0.69**	**−0.69**	−0.25	0.17	**−0.66**	0.01*
Length heterogeneity I											0.25	−0.14	0.41	−0.48	−0.23	0.45	−0.34	0.1*
Weight heterogeneity II												0.29	0.18	−0.25	0.1	0.18	−0.28	−0.23*
Length heterogeneity II													−0.41	0.31	0.31	0.06	0.18	−0.12*
Length at hatching														**−0.97**	−0.38	0.06	**−0.78**	0.31*
Yolk sac volume															0.32	−0.04	**0.82**	−0.34*
Activity I																−0.4	0.49	**−0.76***
Inter-individual distances I																	0.16	0.57*
Activity II																		-0.31*
